# Targeting Shank3 deficiency and paresthesia in autism spectrum disorder: A brief review

**DOI:** 10.3389/fnmol.2023.1128974

**Published:** 2023-02-09

**Authors:** Min Huang, Qi Qi, Tao Xu

**Affiliations:** ^1^Department of Anesthesiology, Sixth People’s Hospital Affiliated to Shanghai Jiao Tong University School of Medicine, Shanghai, China; ^2^Department of Anesthesiology, Suzhou Hospital of Anhui Medical University, Suzhou, China

**Keywords:** autism spectrum disorder, ASD, synapse, Shank3, treatment

## Abstract

Autism spectrum disorder (ASD) includes a group of multifactorial neurodevelopmental disorders characterized by impaired social communication, social interaction, and repetitive behaviors. Several studies have shown an association between cases of ASD and mutations in the genes of SH3 and multiple ankyrin repeat domain protein 3 (SHANK3). These genes encode many cell adhesion molecules, scaffold proteins, and proteins involved in synaptic transcription, protein synthesis, and degradation. They have a profound impact on all aspects of synaptic transmission and plasticity, including synapse formation and degeneration, suggesting that the pathogenesis of ASD may be partially attributable to synaptic dysfunction. In this review, we summarize the mechanism of synapses related to Shank3 in ASD. We also discuss the molecular, cellular, and functional studies of experimental models of ASD and current autism treatment methods targeting related proteins.

## 1. Introduction

### 1.1. Autism

Autism spectrum disorder (ASD) is a term widely used to describe multiple multifactorial neurodevelopmental (Sharma et al., 2018) and grouping disorders (Becerra et al., 2014), including autism (Jiang and Ehlers, 2013), Asperger’s syndrome (Berkel et al., 2018), and no other general developmental disorders (Thapar et al., 2017). However, the updated diagnostic criteria for ASD mainly focus on two core areas: social communication disorder and alternative interests/repetitive behaviors (Kales et al., 2015; Kodak and Bergmann, 2020; Srivastava et al., 2021).

The prevalence of ASD has increased steadily (Kincaid et al., 2017; Hyman et al., 2020). Genetic factors, parental history of mental illness, premature birth, and oxygen exposure to psychotropic drugs or pesticides are associated with a higher risk of ASD (Kincaid et al., 2017; Masi et al., 2017; Crump et al., 2021). Various scales, such as the Childhood Autism Rating Scale (CARS; Moon et al., 2019), Childhood Autism Spectrum Disorder Observation (ASD-OC; Neal et al., 2012), and Developmental, Dimensional, and Diagnostic Interview (3Di; Randall et al., 2018), can be used to assess abnormal behaviors and symptoms in ASD efficiently. Nearly 75% of patients with ASD have coexisting mental illnesses or complications, including ADHD, epilepsy, anxiety, bipolar disorder, depression, Tourette’s syndrome, and inpatient diseases (Tuchman et al., 2010; Doshi-Velez et al., 2014).

Pharmacological and non-pharmacological interventions are used for ASD treatment (Sharma et al., 2018). Existing drug treatments, including psychostimulants, atypical antipsychotics, antidepressants, and α-2 adrenergic receptor agonists, can partially relieve the core symptoms of ASD and control comorbidities (Aman, 2004; Muskens et al., 2017). Moreover, non-pharmacological interventions, including music therapy and cognitive and social behavioral therapies, improve the social interaction and oral communication of patients with ASD (Sharma et al., 2018; Esposito et al., 2020). The combined use of vitamins, herbs, nutritional supplements, and behavioral therapy improves ASD symptoms; however, the specific efficacy needs further investigation (Guang et al., 2018; Sharma et al., 2018).

### 1.2. Shank3

Shank (also known as ProSAP) protein has three main subtypes, Shank1, Shank2, and Shank3, with similar structural domains: N-terminal ankyrin repeats, an Src homology 3 (SH3) domain, a PSD-95/Discs large/ZO-1 (PDZ) domain, an extended proline-rich region, and a sterile alpha motif (SAM) domain (Saupe et al., 2011; Costales and Kolevzon, 2015; Varghese et al., 2017; Sgritta et al., 2019).

As the main scaffold protein of excitatory synaptic PSD, Shank protein interacts with more than 30 types of postsynaptic proteins through these domains. These domains are critical for synapse formation, glutamate receptor transport, and neuronal signal transmission (Monteiro and Feng, 2017; Orefice et al., 2019). Shank3 is encoded by the *SHANK3* gene located on chromosome 22q13.3. The 22q13.3 deletion syndrome [also known as Phelan-McDermid syndrome (PMS)] is found and characterized by marked developmental deterioration (Phelan et al., 2001; Phelan and McDermid, 2012). More than 50% of patients with PMS have identified *SHANK3* abnormalities, including complete deletions, insertions, splicing mutations, and point mutations (Durand et al., 2007; Gauthier et al., 2009; Boccuto et al., 2013). Mutations in *SHANK3* are estimated to occur in 1–2% of people with autism and intellectual disabilities, while mutations in *SHANK1* and *SHANK2* are less common (Boccuto et al., 2013; Leblond et al., 2014; Roberts et al., 2014). Shank3 deficient neurons showed reduced overall expression levels of PSD protein, including GKAP, Homer1b/c, AMPAR subunit GluA1, and NMDAR subunit NR2A (Wang et al., 2011; Peça et al., 2011a). The disruption of the interaction and connection between Shank3, GKAP, and Homer1b/c may cause the redistribution and disruption of the activity-dependent GluA1 subunit. Consequently, posterior tonicity increases, and hippocampal LTP decreases (Wang et al., 2011). In patients with PMS, neurons induced by pluripotent stem cells (iPSCs) showed significantly impaired NMDAR and AMPAR-mediated synaptic transmission (Shcheglovitov et al., 2013; Speed et al., 2015; Qin et al., 2018; Tatavarty et al., 2020). Shank3-deficient mice showed repetitive self-harm and social interaction defects. In addition, mEPSC frequency and amplitude were significantly reduced, indicating a reduction in the number of functional synapses and a decrease in the postsynaptic responses of available synapses (Peça et al., 2011b; Lee et al., 2021). In contrast, overexpression of Shank3 resulted in a sharp increase in the amplitude of AMPAR-mediated NMDAR and NMDAR-mediated EPSC and a high frequency of AMPAR-mediated mEPSC (Arons et al., 2012).

### 1.3. Risk factors of ASD

The prevalence of ASD in boys is four to five times that in girls (Christensen et al., 2018). Patients with genetic and chromosomal diseases tend to show more symptoms of ASD (Ogata et al., 2014). About 10% of children with ASD also have Down syndrome or Fragile X syndrome (DiGuiseppi et al., 2010; Auerbach et al., 2021). The psychiatric history of biological parents, especially the history of schizophrenia and affective disorder, is associated with an increased incidence of ASD (Jokiranta et al., 2013). Fetal exposure to pesticides is associated with a decrease in infant weight and length, delay in psychomotor development, and high risk of autism (Landrigan, 2010). In addition, epidemiological studies have shown that exposure of pregnant mothers to viral or bacterial infections, especially in the first trimester or middle of pregnancy, promotes the mother’s immune activation (MIA) and increases the risks of children’s neuropsychiatric disease, including ASD (Estes and McAllister, 2016). MIA is related to an increase in neuroinflammatory cytokines, abnormal expression of synaptic proteins, and abnormal development of synaptic connections, all of which may contribute to the pathophysiology of ASD (Pendyala et al., 2017). Consuming psychotropic drugs during pregnancy is considered a risk factor for autism. Several studies have demonstrated that prescribing antidepressants to pregnant women increases the risk of autism moderately (Gidaya et al., 2014; Siu and Weksberg, 2017). Certain limitations, including failure to carefully adjust the mother’s psychiatric history (Hoover and Kaufman, 2018; Bai et al., 2020), genetic susceptibility to ASD (Pardo and Eberhart, 2007; Wei et al., 2021), and variable molecular and clinical effects of different antidepressants (McCarthy et al., 2016; Soler et al., 2018; Johannessen et al., 2019), may lead to differences in findings of current and repeated studies. However, a recent 12-year study (including full-term live births by mothers who received antidepressants during pregnancy) concluded that the period of antidepressant use did increase the risk of autism in children (Boukhris et al., 2016; Modabbernia et al., 2017). A previous pilot study evaluated medical screening results at the time of referral for children and adolescents with different mental disorders. They found newly developed somatic functions in 56% of the subjects (Muskens et al., 2015). These findings include a wide range of medical problems, including weight and height problems (Dhaliwal et al., 2019), high thyroid hormone levels (Needham et al., 2021), dyslipidemia (Panjwani et al., 2020), anemia (Wiegersma et al., 2019), vitamin D and B12 deficiency (Raghavan et al., 2018; Wang Z. et al., 2020), and malformations (Grafodatskaya et al., 2010). Some of these results require consultation with other medical experts. In contrast, other results directly impact daily medical practice, such as adjusting psycho-drug logic therapy and participating in overweight prevention plans (Vannucchi et al., 2014; Mansouri et al., 2020). These studies contribute to improving people’s understanding of the relationship between somatic and mental symptoms in developmental disorders. It points to common genetic pathways and other potential mechanisms that may be involved (Dovey et al., 2019; Frolli et al., 2021). In addition, the simultaneous assessment of medical and psychiatric disorders may be of great value. Clinicians can then relate somatic functions to different diagnostic considerations for medical and psychological intervention (Rodin et al., 2021).

## 2. Shank3 and ASD

### 2.1. Links between Shank3 genes and ASD

**Figure 1:**
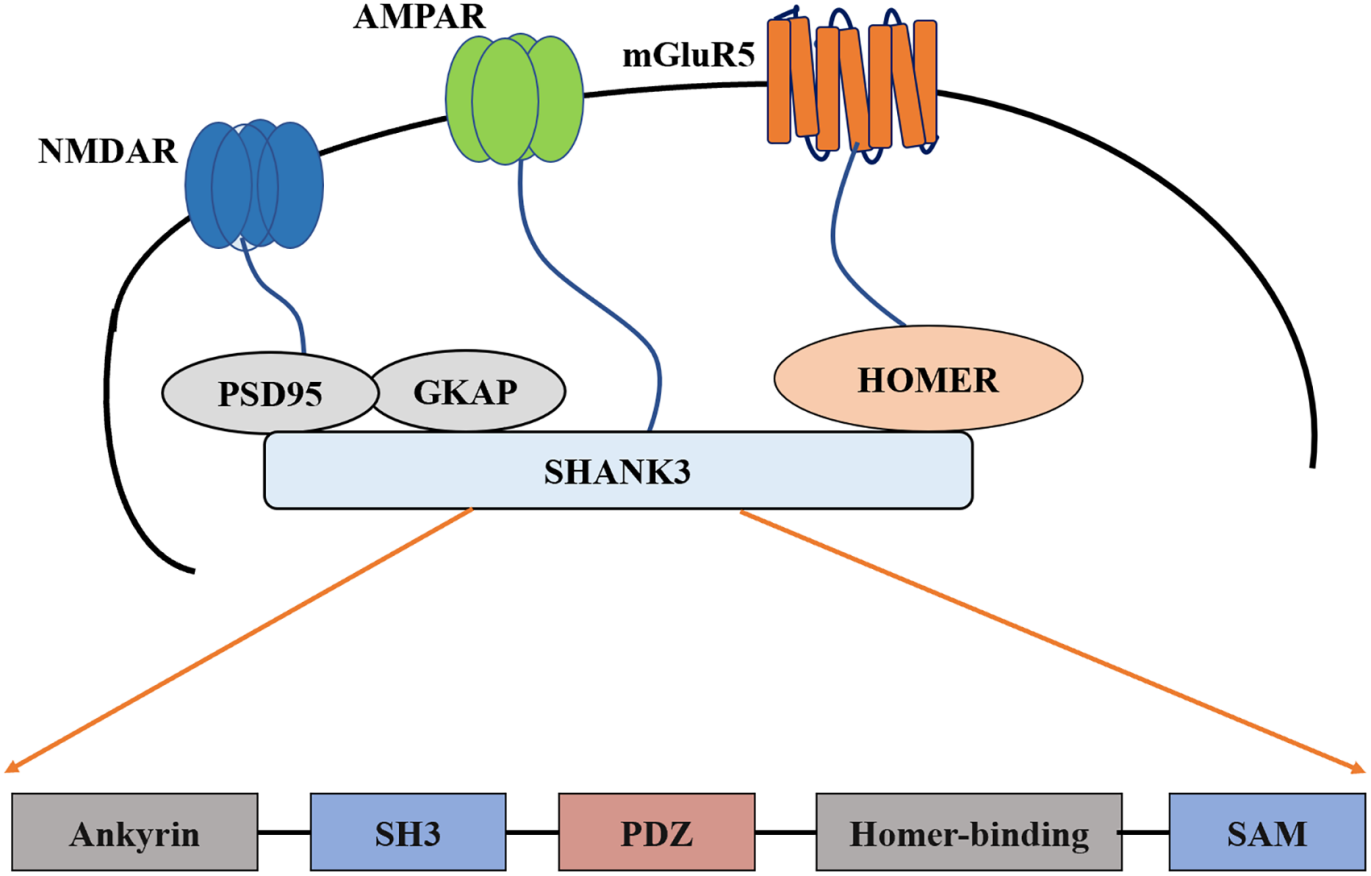
Schematic diagram of postsynaptic density and construction of Shank3. PSD-95, Postsynaptic density-95; SH3, Src homology 3; PDZ, PSD95/DlgA/Zo-1; SAM, Sterile alpha motif; mGluR5, metabotropic glutamate receptor 5; AMPAR, The α-amino-3-hydroxy-5-methyl-4-isoxazolepropionic acid receptor; NMDAR, The N-methyl-D-aspartate receptor; GKAP, guanylate kinase-associated protein.

The *SHANK* gene was first found to be related to neurodevelopmental disorders in the study of PMS (Costales and Kolevzon, 2015; Frank, 2021). PMS is a neurodevelopmental disorder caused by the deletion of 22q13.3, characterized by autistic behavior, hypotonia, and continuous or even nonexistent speech (Burdeus-Olavarrieta et al., 2021). Genome rearrangements in patients with PMS include deletions, chromosomal, mesenchymal deletions, and unbalanced translocations (Bonaglia et al., 2011; Leblond et al., 2014; Tammimies, 2019). In almost all reported cases (Wilson et al., 2003; Wilson et al., 2008), the loss of Shank3 was observed. These cases support the theory that PMS symptoms are caused by the loss of the SHANK3 haplotype (Phelan and McDermid, 2012) or complete *SHANK3* gene on chromosome 22 (Grabrucker et al., 2011a,b). In addition to the symptoms of autism, genetic screening of patients with ASD who have not yet been diagnosed with PMS also revealed many *SHANK3* mutations (Durand et al., 2007; Moessner et al., 2007; Gauthier et al., 2009; Boccuto et al., 2013; Leblond et al., 2014). These mutations include small deletions, nonsense mutations, breakpoints, and missense mutations. A meta-analysis study found that mutations or disruptions in the SHANK gene family accounted for about 1–2% of all patients with ASD (Leblond et al., 2014; Zhou et al., 2016). The degree of related mutations and cognitive impairment between SHANK1-3 also differs: patients with *SHANK3* mutations suffer more cognitive impairment than those with *SHANK1* or *SHANK2* mutations (Qin et al., 2022). In addition, patients with *SHANK3* mutations have severe cognitive deficits (Chevallier et al., 2012; Zhou et al., 2016). These findings together indicate that the common neurobiological effects shared by all members of the *SHANK* gene family may be related to the pathophysiology of ASD. Furthermore, the degree of cognitive impairment in ASD may be due to mutations in the *SHANK* family members, the most significant being *SHANK3* (Chevallier et al., 2012; Baum et al., 2015; Monteiro and Feng, 2017; Banker et al., 2021). The difference in the severity of symptoms can be determined by the mutation of the specific *SHANK* gene (Jiang and Ehlers, 2013; Gong and Wang, 2015; Li and Pozzo-Miller, 2020). The results of the study suggest that mutations in the *SHANK3* gene are the primary cause of ASD and that the expression of the other two remaining *SHANK* subtypes can (or cannot) compensate for its loss (Mashayekhi et al., 2021; Salomaa et al., 2021). Family mutations are comprehensive and should be screened in clinical practice. Many human *SHANK3* mutations map to exon 21 and are associated with moderate to severe intellectual disability (D'Antoni et al., 2014; Mossa et al., 2021; Purushotham et al., 2022). Mutations in the pro-domain region of exon 21 were not associated with altered pathophysiology (Shi et al., 2017; Moutin et al., 2021). One possible explanation is that exon 21 is present in most *SHANK3* isotype surrogates (Moutin et al., 2021). Therefore, mutations in this exon may have more severe effects (Speed et al., 2019). Given the nature of these genes in ASD, it becomes imperative to understand their usual role in synapses and how mutations disrupt them (Figure 1).

### 2.2. Neurobiological characteristics of ASD models with alterations in the Shank gene

The expression of different Shank3 subtypes varies with different brain regions and ages (Wang et al., 2014b; Vyas et al., 2021), complicating the analysis and establishment of animal models. Studies on various mouse models of Shank3 heterozygous deletion have recorded changes in the transport of glutamate transmitters or synapses (Yi et al., 2016; Lutz et al., 2020). For example, the Shank3 (Shank3α) model, by deleting exons 4–9, shows reduced immunoreactivity of the glutamate receptor 1 in hippocampal CA1 (Bozdagi et al., 2010; Epstein et al., 2014). In Shank3B^−/−^ mice, gene deletion of two consecutive Shank3 isoforms (Shank3α and β) increases the length and complexity of dendrites and decreases the synaptic density of spinal neurons in the striatum and PSD length and thickness (Peça et al., 2011a; Balaan et al., 2019; Rendall et al., 2019). Purkinje cells of Shank3^+/ΔC^ mice have Shank3 C-terminal deletions, although the density does not change, and they have a robust dendritic complexity in the body and reduced synaptic density (Kloth et al., 2015; Zhu et al., 2018). The expression of NMDA receptors in the PFC of these mice was reduced; however, the density of synapses did not change (Duffney et al., 2015). The dendrites on the CA1 neurons of Shank3^ΔC/ΔC^ mice reduced LTP and NMDA/AMPA ratios in the hippocampus; however, their complexity or synapse density did not change (Bangash et al., 2011; Kouser et al., 2013). In a KO mouse model (Shank3Δe4–22^−/−^ mice) that eliminated all Shank3 subtypes, the PSD length and thickness of the striatum were reduced (Pagliardini et al., 2005; Bey et al., 2018). Restoring Shank3 expression in adult Shank3-deficient mice can reverse the destruction of dendritic spines in the striatum and stimulate synaptic function (Peça et al., 2011a; Mei et al., 2016; Hsueh, 2019). The above findings confirmed the role of Shank3 in mobilizing multiple glutamate receptor assemblies at the PSD and synaptic signal substitution and dynamics (Jeong et al., 2021). Although the loss of heterozygosity in Shank3 is more representative of the defects observed in human PMS, the researchers still focused on the homozygous Shank3 KO (Han et al., 2016; Yi et al., 2016; Jacot-Descombes et al., 2020).

Based on the molecular and physiological abnormalities in SHANK3-deficient animals (Delling and Boeckers, 2021), various pharmaceutical compounds have been proposed as possible therapeutic options for SHANK3-related neuropsychiatric disorders (Wang X. et al., 2016; Vicidomini et al., 2017). For example, modulation of glutamate receptors may be beneficial, as AMPAR, NMDAR, or GRM5 hypofunction may lead to excitation/inhibition imbalance, contributing to ASD-like phenotypes in SHANK3-mutant mice (Guo et al., 2019; Rhine et al., 2019; Nuzzo et al., 2020).

## 3. Comorbidity of Shank3 deficiency and paresthesia

Hypersensitivity, hyposensitivity, or abnormal interest in sensory stimuli are common features of patients with ASD (Lord et al., 2020) and PMS, who usually show increased pain tolerance (Zencica et al., 2010; Soorya et al., 2013). Abnormal sensory function in *SHANK3*-deficient rodents can be analyzed in various ways. The most used paradigm focuses on the perception of injury or somatic sensory function. Examples of the methods used include the tail-flick, Frey, and hot plate tests. However, other methods have also been investigated, including auditory, olfactory, visual, vestibular function, and sensorimotor gating (Bruno et al., 2021). In rat models, exons 4–22 (Han et al., 2013; Drapeau et al., 2018), exon 11 (Vicidomini et al., 2017), exon 21 (Kouser et al., 2013), and exons 11–21 (Song et al., 2019) describe general somatic dysfunction, especially low pain sensitivity. Targeted destruction of SHANK3 in tail embryo cells, somatosensory neurons [exon 13–16 (Orefice et al., 2019)], and cells expressing SCN10A [commonly known as Nav1.8, exon 4–22 (Han et al., 2013)] can induce somatosensory dysfunction. Two studies examining the constitutive SHANK3 defect model exon 13–16 (Chen et al., 2020; Kabitzke et al., 2020) described somatosensory dysfunction related to light touch stimulation. In contrast, in other studies, the somatosensory function examined by heat/injury perception was not affected in these mice (Dhamne et al., 2017; Schroeder et al., 2017). Additionally, there is mixed evidence regarding the mouse model of exons 4–9 (Yang et al., 2012; Orefice et al., 2019), while the somatic sensory function of mice with exon 8 appears unaffected (Yoo et al., 2019).

These previous studies suggest that ASD subjects express their pain perception through various behaviors, including typical reactions similar to those seen in the general population. Crying, shouting, protecting the painful parts, or seeking comfort are examples. However, the behaviors are more specific and less easily recognized as pain-related behaviors (making specific sounds, playing posture, etc.; Dubois et al., 2017). Despite these findings, the version of the Diagnostic and Statistical Manual of Mental Disorders (DSM 5) still mentions sensitivity to pain as a diagnostic standard and uses the term “obvious pain indication.” Pain sensitivity is a critical issue for patients with autism because they experience pain more frequently than others (Gillberg and Coleman, 1996; Lee et al., 2008). The sensitivity of patients with autism to pain may affect their medical pain management. This may lead to an insufficient treatment or a lack of evaluation. Therefore, this assumption needs to be tested.

Although some adults with ASD may also experience reduced pain sensitivity, lack of pain perception may not be considered a classic characteristic of ASD populations. To understand the pain in ASD and explain its lack of reactivity, it is necessary to focus on multiple components of pain rather than just the mechanism of pain and consider pain as part of a broader conceptual framework (Hadjistavropoulos and Craig, 2002; Dubois et al., 2010; Rattaz et al., 2013; Moore, 2015). Conceptualizing pain expression from the perspective of multiple factors requires individual pain management strategies and evaluation according to the clinical characteristics of patients with autism.

## 4. Treatment of ASD and paresthesia by targeting Shank3

Current treatment options for ASD include pharmacological and non-pharmacological interventions. Pharmacological interventions include psychostimulants, atypical antipsychotics, antidepressants, alpha-2 adrenergic receptor agonists, NMDA receptor antagonists, and antiepileptic mood stabilizers (Aman et al., 2008; Costales and Kolevzon, 2015; Bicker et al., 2021). This section focuses on the safety and tolerability profiles of the main strategies used to treat children and adults with ASD (Table 1).

**Table 1:** Targets in Shank3 and treatment effects.

Target	Edit	Related factors	Effect
PDZ domain	Inverted	Homer, GluN2A, GluN2B and GluR2	Repetitive modification, anxiety, social interaction deficits and impaired motor coordination
	Restored in adulthood		All the changes restored
Full length Shank3 gene	Knock-out	mGluR5, AMPAR and NMDAR	Repetitive modification, anxiety, social interaction deficits and impaired motor coordination
	Re-expression in adulthood		Reduce repetitive behaviors and social interaction defects, but cannot relieve anxiety or motor coordination defects,
Exon 11	Knock-out	RAC1 and PAK	Social behavior defects
Exon 6 and 7	Knock-out	GABA	Spatial memory deficits

PDZ, PSD95/DlgA/Zo-1; GluN2A, NMDA receptor 2A; GluN2B, NMDA receptor 2B; GluR2, Glutamate ionotropic receptor AMPA type subunit 2; mGluR5, metabotropic glutamate receptor 5; AMPAR, The α-amino-3-hydroxy-5-methyl-4-isoxazolepropionic acid receptor; NMDAR, The N-methyl-D-aspartate receptor; RAC1, Rac Family Small GTPase 1; PAK, p21-activated kinase; GABA, gamma-aminobutyric acid.

Because the genome of human mutations associated with ASD is very complex, animal models are the basis for studying specific mutations and establishing beneficial genotype-cell phenotypes (De Rubeis et al., 2018; Zhou et al., 2019; Reed et al., 2020). New approaches to preclinical animal studies should take into account evidence that certain therapeutic windows affect specific circuits and associated behavioral phenotypes, but also potentially reopen those critical plasticity periods to enhance therapeutic effectiveness. In particular, the advantage of the SHANK3 gene is that different mouse models can be designed, including different Shank3 gene deletions or failures (Engineer et al., 2018; Poleg et al., 2021). In addition, the SHANK3 protein has a precise location in the glutamatergic synapse. The study of the Shank3 gene is less complicated than that of other autism-related genes (Uchino and Waga, 2015; Tao-Cheng et al., 2016; Wang L. et al., 2020; Golden et al., 2021). Since autism is a neurodevelopmental disorder (symptoms appearing before age three; Lord et al., 2000; Bonaglia et al., 2001), one of the critical questions in autism research is whether the symptoms are reversible in adulthood. Recently, in a study, mice with Shank3-KO (with the deletion of the PDZ domain) had an inverted PDZ domain so that they could be repositioned at any point in the growth phase to re-express the Shank3 gene (Jaramillo et al., 2016, 2017; Jacot-Descombes et al., 2020). This gene design is crucial because it keeps the Shank3 gene under the control of its endogenous genome and avoids the expression of the SHANK3 gene at a non-physiological level, which may cause potential confusion (Saré et al., 2020). These Shank3-KO mice had defects in neurotransmission in the striatum. As a result, the striatum’s synapse density and the levels of essential PSD proteins (SAPAP3, Homer, GluN2A, GluN2B, and GluR2) were reduced. Behaviorally, these Shank3-KO mice showed repetitive self-harm modification, anxiety, social interaction deficits (decreased frequency and duration of social interaction), and impaired motor coordination. By restoring the expression of the Shank3 gene, all these changes could be restored in adulthood (Mei et al., 2016; Guo et al., 2019; Lee et al., 2021).

Re-expression of Shank3 in adulthood can reduce repetitive self-harm behaviors and social interaction deficits but not anxiety or motor coordination defects. Accordingly, this re-expression can only save a portion of the behavioral manifestations of autism (Grabrucker et al., 2011a; Peça et al., 2011a; Tai et al., 2020). A similar study showed that early postpartum intervention could improve irreversible behavioral defects in adulthood (Jin et al., 2018; Bukatova et al., 2021). Therefore, this phenomenon emphasizes the unique performance of SHANK3 expression at specific developmental stages and throughout life. Relying on the emergence of novel gene editing methods (such as CRISPR; Liu et al., 2018; Tu et al., 2019; Chiola et al., 2021), repairing the SHANK3 gene in adulthood can alleviate some synaptic and behavioral disorders related to SHANK3 mutations. Although there are still technical limitations to the genetic manipulation of mature neurons in the fully adult brain, recent research has promoted the application of CRISPR to adult brain repair (Swiech et al., 2015; Lee et al., 2017). More importantly, this work shows the possibility of treating patients with SHANK3 mutations or deletions during adulthood (either pharmacologically or through future genetic modification methods).

Some studies have shown that the re-expression of the Shank3 gene in the brain leads to a complete reversal of the expression of the SHANK3 protein. Previous studies have verified this result (Speed et al., 2015). However, we cannot conclude that this biochemical rescue leads to the rescue of some behaviors or synaptic phenotypes in mutants. Transgene seems to have complex effects on wild-type mouse synaptic transmission (Han et al., 2013; Lin R. et al., 2021). Nevertheless, some scholars have successfully replicated the previous behavioral and electrophysiological findings in Shank3 mutant mouse models (Kouser et al., 2013; Speed et al., 2015).

In summary, these studies indicate that restoring Shank3 levels or downstream signals in adults may be one of the therapeutic ways to alleviate certain synaptic and behavioral disorders associated with Shank3 mutations (Bariselli et al., 2016; Jaramillo et al., 2020; Reichova et al., 2020). As downstream mediators and proteins related to the Shank3 network are regulated, two groups have recently studied mGluR5 and Homer as potential therapeutic targets in ASD (Kouser et al., 2013; Wang X. et al., 2016; Vicidomini et al., 2017; Huang et al., 2021). Using complete Shank3-KO mice (Wang X. et al., 2016), they demonstrated that inhibition of mGluR5 activity could reduce excessive licking, while positive agonists of mGluR5 aggravated self-licking. In another study (Vicidomini et al., 2017), the pharmacological enhancement of mGluR5 activity improved repetitive behavior and rescued other behavioral defects in Shank3-KO mice (Verpelli et al., 2011; Lin et al., 2014). Although these studies may seem contradictory, the mGluR5 positive agonist CDPPB exacerbated self-modification in these studies. These results are based on different Shank3-mutant transgenic mice, which can lead to different results. However, the findings from both studies are consistent in other respects. According to another study (Vicidomini et al., 2017), pharmacological activation of mGluR5 activity alleviated functional (NMDA-induced synaptic membrane depolarization; Arvanov and Wang, 1997; Pisani et al., 2001) and behavioral defects (social interaction and Morris water maze; Wang Y. et al., 2016) in mice with exon 11 deletion. Another study reported that the cortical actin filaments of mice lacking exon 11 were significantly reduced (Duffney et al., 2015). This was attributed to the reduced activity of RAC1 and PAK, as well as the enhanced activity of cofilin (the main factor involved in actin depolymerization; Al-Ayadhi and Halepoto, 2013; Sarowar and Grabrucker, 2016; Yi et al., 2016). This suggests that actin modulators may be another potential molecular target for treating ASD. The increase of RAC1 activity in the PFC of these mice improved their social behavior defects and NMDAR function (Park et al., 2003; Duffney et al., 2013, 2015). In contrast, inhibition of PAK or RAC1 function resulted in social behavior defects and dysregulation of NMDAR function in wild-type mice (Bennett and Lagopoulos, 2014; Lin Y. et al., 2021). The use of drugs in Shank3 mutant mice to reverse their symptoms highlights the potential target pathway. NMDA hypofunction is a potential mechanism of ASD behavior (Won et al., 2012; Lee et al., 2015). Social interaction could be improved by treating mice with CDPPB (probably due to the enhancement of NMDAR function through mGluR5 activation; Won et al., 2012). In recent years, many studies have investigated NMDA-dependent inhibitors, demonstrating their therapeutic efficacy in ASD (King et al., 2001; Minshawi et al., 2016; Wink et al., 2017). The mechanism of the NMDAR antagonist, ketamine, acting on the nervous system, significantly overlaps with the pathophysiological theory of ASD, including destroying synaptic connections and neuronal networks (Wang et al., 2014a,b; Krüttner et al., 2022). However, despite the broad interest throughout psychopharmacology research, ketamine has not been explored in clinical trials of ASD. Furthermore, mice lacking exons 6 and 7 have impaired GABAergic neurotransmission (Orefice et al., 2016; Lim et al., 2017). Collectively, these studies suggest that NMDAR hypofunction leads to specific ASD-like phenotypes in Shank-mutant mice, and other related molecular targets may be used to regulate NMDAR activity. Direct gene targeting in humans appears to be a future treatment option for some types of ASDs, as the recent success of many techniques related to detecting and treating genetic disorders may provide the necessary tools.

## 5. Conclusion

Although Shank3 mutation is heterozygous in humans, the analysis and identification of Shank3 homozygous mutant mice are imperative for understanding the physiological role of Shank3 and its functional consequences. In addition, the mutation has destructive effects. In terms of the treatment window, the earlier the treatment is administered, the better the outcome. However, interventions in adulthood may still be useful for reducing some of the symptoms associated with SHANK3 mutations. It is necessary to carefully analyze the specific phenotype of a genotype before trying a drug alone. Exploring the *SHANK3* gene may help uncover some of the neurobiological aspects of autism.

## Author contributions

MH and TX performed the conceptualization. MH and QQ searched the literature and prepared the draft. All authors contributed to the article and approved the submitted version.

## Funding

This work was supported by grant from the Natural Science Foundation of China (82171486), Natural Science Foundation of Shanghai to TX (21ZR1448400), the Interdisciplinary Program of Shanghai Jiao Tong University to TX (YG2021ZD23), and General Science Foundation of Shanghai Sixth People’s Hospital to TX (YNMS202114).

## Conflict of interest

The authors declare that the research was conducted in the absence of any commercial or financial relationships that could be construed as a potential conflict of interest.

The reviewer ZQ declared a shared parent affiliation with the authors to the handling editor at the time of review.

## Publisher’s note

All claims expressed in this article are solely those of the authors and do not necessarily represent those of their affiliated organizations, or those of the publisher, the editors and the reviewers. Any product that may be evaluated in this article, or claim that may be made by its manufacturer, is not guaranteed or endorsed by the publisher.
